# Comparison of Intake of Food Groups Based on Dietary Inflammatory Index (DII) and Cardiovascular Risk Factors in the Middle-Age Population of Lower Silesia: Results of the PURE Poland Study

**DOI:** 10.3390/antiox12020285

**Published:** 2023-01-27

**Authors:** Alicja Szypowska, Bożena Regulska-Ilow, Katarzyna Zatońska, Andrzej Szuba

**Affiliations:** 1 Department of Dietetics, Wroclaw Medical University, 50-556 Wroclaw, Poland; 2 Department of Dietetics and Bromatology, Wroclaw Medical University, 50-556 Wroclaw, Poland; 3 Department of Population Health, Wroclaw Medical University, 50-368 Wroclaw, Poland; 4 Department of Angiology, Hypertension and Diabetology, Wroclaw Medical University, 50-556 Wroclaw, Poland

**Keywords:** cardiovascular diseases, cardiovascular risk, dietary inflammatory index, diet, PURE study

## Abstract

Chronic inflammation is involved in the pathogenesis of many non-infectious diseases, including cardiovascular diseases (CVD), a leading cause of death in Europe. The aim of the study was to assess the inflammatory potential of the diets of participants enrolled in the Polish arm of the Prospective Urban and Rural Epidemiological (PURE) study, evaluate the association between the dietary inflammatory index (DII) score with the dietary content, and to determine the correlation of DII score with selected anthropometric parameters and biochemical risk factors for CVD. Diets were assessed with the Food Frequency Questionnaire (FFQ). Among participants with pro-inflammatory diets, we reported higher mean values of triglycerides (TG), fasting glucose (FG), atherogenic index of plasma (AIP), and the Castelli’s risk index (CRI) in the group of men and women, and higher waist circumference (WC) and waist-to-hip ratio (WHR) in the group of women. Pro-inflammatory diets were associated with higher intake of refined grains, sweets, juices, red meat, high-fat cheese and cream, alcohol, fats except for vegetable oils, potatoes, sugar and honey, French fries, fried fish, and processed/high-fat poultry. Moreover, study participants with pro-inflammatory diets consumed more milk, low-fat dairy, and eggs associated with unhealthy dietary habits, but this should not be considered as an independent CVD risk factor. Anthropometric and biochemical outcomes were more favorable among study participants who consumed more vegetables, fruits, nuts, seeds, raisins, pulses, low-fat poultry, and tea. However, association of beverage consumption with dietary inflammatory potential requires further study.

## 1. Introduction

Chronic inflammation is involved in the pathogenesis of many non-infectious diseases, including cardiovascular diseases (CVD) [1], a leading cause of death in Europe [2]. Based on 2020 data, CVD in Poland was associated with 41% of all deaths among women and 33% among men [3]. Preventing CVD on individual and population levels is one of the main challenges for medical personnel and policy makers [4,5]. The European Society of Cardiology (ESC) guidelines on CVD prevention put a great emphasis on non-pharmacological interventions, i.e., screening for CVD markers and risk factors, maintaining adequate exercise, diet modifications, weight reduction, identifying smokers and providing advice on smoking cessation, diagnosing, and appropriate treatment of hypertension, hyperglycemia, and hyperlipidemia [4].

Diet is one of the most important lifestyle factors in the development of CVD as it can increase systemic inflammation [5]. The Mediterranean diet is the best studied diet in the world. It has been found that preventive use of a Mediterranean diet (or a Dietary Approaches to Stop Hypertension-DASH diet) reduces the risk factors for CVD [4,6]. Results of epidemiological studies showed that healthy dietary patterns, i.e., a high intake of fruits, vegetables, legumes, whole grains, fish, low-fat dairy, and foods rich in antioxidants (omega-3 fatty acids, flavonoids) is associated with a reduction of low-grade inflammation, oxidative stress, and improved endothelial function [7,8,9,10]. On the other hand, consumption of the Western-pattern diets, which is characterized by high intakes of highly processed foods, simple carbohydrates, refined grains, red processed meats, and foods rich in saturated fatty acids and sodium, has been associated with chronic inflammation [11,12].

Dietary inflammatory index (DII) is a scoring algorithm based on an extensive review of the literature from 1950 to 2010. In total, DII authors reviewed 1943 articles with 45 selected food parameters. Dietary inflammatory index score, developed to indicate the inflammatory potential of a diet, can be associated with systemic inflammation [13,14] and CVD indicators [15,16,17]. The DII authors evaluated the association of dietary components with six inflammatory biomarkers i.e., interleukin-1β (IL-1β), IL-4, IL-6, IL-10, tumor necrosis factor alpha (TNF-α), and C-Reactive Protein (CRP). The inflammatory potential for each food parameter was scored according to whether it increased (+1), decreased (−1), or had no effect on the six inflammatory biomarkers [18].

Shivappa et al. [19] reported a 36% increased risk of CVD incidence and mortality among individuals with the highest DII scores (pro-inflammatory diet) compared to individuals with the lowest DII scores (anti-inflammatory diet). Despite the well-studied association between inflammatory biomarkers and chronic inflammation-related disease endpoints, the relationship between DII and intermediate biomarkers of cardiometabolic health remains largely unknown.

A recent meta-analysis showed that adherence to a pro-inflammatory dietary pattern had a significant positive association with 27 (71%) of the included health outcomes (*p* < 0.005), however Class I evidence was identified only for myocardial infarction along with a higher, i.e., more pro-inflammatory DII score. The strength of evidence was limited for most health outcomes so there is a need for further research [20].

Recently, only a few studies have analyzed the association between the DII score and cardiometabolic markers in the European population [15,16,17]. Given the fact that Poland was classified as a country at high-risk of CVD [4], it is of utmost importance to use non-invasive, concrete, rapid tools to identify individuals at high risk of developing CVD [21]. Besides, detailed identification of specific food groups according to their inflammatory potential seems relevant to formulate appropriate dietary recommendations. The Atherogenic index of plasma (AIP) and Castelli’s Risk Index (CRI) have been developed to estimate the risk of CVD [21,22]. A recent study including data from the National Health and Nutrition Examination Survey indicated that AIP may be a stronger predictor of cardiovascular risk than individual cholesterol risk factors [23].

The aim of the study was to assess the inflammatory potential of the diets of participants enrolled in the Polish arm of the Prospective Urban and Rural Epidemiological (PURE) study, evaluate the association between the DII score with the dietary content, and to determine the correlation of DII score with selected anthropometric parameters and biochemical risk factors for CVD.

## 2. Materials and Methods

### 2.1. Study Population

The PURE study is an international cohort study which at baseline involved 153,996 adults from 17 countries with different income levels. The Polish participants of the PURE study were low-, middle- and high-income Polish adults. The inclusion criteria for the survey were: age between 35–70 and a permanent place of residence in an urban or rural area of the Lower Silesia in Poland. Individuals were recruited to the Polish arm of the PURE study through the radio and television announcements. The aim of the study was to calculate the association between the urbanization level and CVD prevalence and risk factors. The main results of the study have been previously published [24,25]. The first stage of the study was conducted between 2007 and 2009 and included a food frequency questionnaire (FFQ), blood draw, blood pressure measurements, spirometry, and anthropometric measurements. There was a total of 2039 study participants. Individuals who did not meet the criterion of adequate dietary energy intake (for men < 800 kcal, >4200 kcal, for women < 600 kcal, >3500 kcal) were excluded. The above criteria were established in accordance with recommendations [26]. In addition, participants were excluded from the study due to missing data for more than one variable. Finally, a total of 1791 individuals were included in the study.

### 2.2. Measurement of Cardiovascular Risk Factors

The concentrations of fasting glucose (FG), triglycerides (TG), high-density lipoprotein (HDL-C), and total cholesterol (TC) were measured in venous blood samples. SPINREACT enzymatic test kit (Sant Esteve De Bas, Girona, Spain), was used to measure HDL-C and TG concentrations. If participants had a TG concentration lower than 400 mg/dL, low-density lipoprotein cholesterol (LDL-C) was calculated based on the Friedewald equation (LDL-C = TC − HDL-C − TG/5). Fasting glucose was measured after an overnight fasting period with the Ascensia ENTRUST Glucometer kit (Bayer, Germany). The above variables were expressed in mmol/L except for FG, which was expressed in mg/dL. Systolic and diastolic blood pressure was measured with a certified automatic blood pressure monitor (Omron HEM-711 IntelliSense, Tokyo, Japan) and expressed in mmHg. Study participants were recommended to rest for 5 min before blood pressure measurement. In the PURE study, blood pressure was measured twice. Waist circumference (WC) was measured midway between the lowest rib and the upper iliac crest, with a standard measuring tape, to the nearest 0.5 cm. Height (H) was measured without shoes, with a stadiometer, and recorded to the nearest 0.5 cm. Weight (W) was measured without shoes or outer garments to the nearest 0.1 kg using a body composition monitor Tanita BC-554 (Japan). Body mass index (BMI) was calculated as weight in kilograms divided by height in meters squared, according to the equation: BMI = W(kg)/H^2^(m^2^). Hip circumference was measured at the level of the largest lateral extension of the hips and expressed in centimeters to the nearest 0.5 cm. Waist-to-hip ratio (WHR) was calculated as WC divided by hip measurement.

### 2.3. Atherogenic Lipid Indices

Lipid profile parameters were used to calculate the atherogenic indices (AIP and CRI). The AIP was calculated as log TG (mg/dL)/HDL-C(mg/dL), where results <0.11 indicated low, 0.11–0.21 moderate, and >0.21 increased risk of CVD. Castelli’s risk index was calculated as TC[mg/dL]/HDL-C[mg/dL], where results <3.5 indicated low, 2.5–4.5 moderate, and >4.5 high risk of CVD [27].

### 2.4. Atherogenic Diet Index

To calculate the atherogenicity of daily food rations, we used the polyunsaturated fatty acid (PUFA)g/saturated fatty acid (SFA)g equation. Assuming that dietary intake of PUFAs should not be less than 6% of a person’s total energy consumption and of SFAs up to 10%, the minimum value of the ratio should be 0.6, and the optimal ratio be above 1.0 [28,29].

### 2.5. Dietary Intake Assessment

Participants’ habitual food intake was assessed with the FFQ, which was developed and validated for the population of PURE study Lower Silesia [30]. The frequency of consumption of selected foods was assessed with 10 possible responses: never, less than once a month; 1–3 times a month, once a week, 2–4 times a week, 5–6 times a week, once a day, 2–3 times a day, 4–5 times a day, and >6 times a day. The FFQ, which was country- and culture-specific, asked about the average consumption during the year preceding the survey and assessed the frequency of consumption of 154 food products, which were divided into 27 food groups (Table 1). The nutritional value of diets was calculated using Polish national food composition tables [31]. The “Album of photographs of food products and dishes” by the National Food and Nutrition Institute in Warsaw was used to determine the average size of the consumed portion [32]. The FFQ and its standardization have been described previously [33].

### 2.6. Demographic Factors

Place of residence was classified as rural or urban and education: elementary/unknown, trade, and secondary/high school or university. The International Physical Activity Questionnaire (IPAQ) was used to calculate physical activity and expressed as metabolic equivalent minutes per week. The number of metabolic equivalent (MET)-min/week lower than 600 was considered low, 600–3000—moderate, and above 3000—high [34]. Smoking status was classified into 3 categories: non-smoker, ex-smoker, and current smoker.

### 2.7. Calculation of Dietary Inflammatory Index

The DII is an algorithm developed to categorize various diets according to their inflammatory potential. A modified and updated version of DII calculation designed by Shivappa et al. [18] was used in this study. A detailed description of the updated DII has been previously described [18]. DII was compiled based on an extensive review of the literature from 1950 to 2010. In total, DII authors reviewed 1943 articles with 45 selected food parameters. Authors of DII evaluated the association of dietary components with 6 inflammatory biomarkers (IL-1β, IL-4, IL-6, IL-10, TNF-α and CRP). The inflammatory potential for each food parameter was scored according to whether it increased (+1), decreased (−1), or had no effect on 6 inflammatory biomarkers. Authors of DII calculated the global daily average intake of each dietary food product, along with the standard deviation, based on 11 data sets from around the world (USA, Australia, the Kingdom of Bahrain, Denmark, India, Japan, New Zealand, Taiwan, South Korea, Mexico, and United Kingdom). Dietary intake of the DII components was compared to the standard global as a Z-score, which was achieved by subtracting the standard mean from the amount reported and dividing this value by its standard deviation [18]. Then, this value was converted to a centered percentile score. To achieve a symmetrical distribution with values centered on 0 (null) and bounded between −1 (maximally anti-inflammatory) and +1 (maximally pro-inflammatory), each percentile score was doubled and then ‘1’ was subtracted. The centered percentile values were then multiplied by the overall pro- and anti-inflammatory effect score for each dietary component. Finally, all results were summed. Higher scores indicated that the diet was more pro-inflammatory, and lower DII scores represented a more anti-inflammatory diet. Results ranged from 7.98 (maximally pro-inflammatory) to −8.87 (maximally anti-inflammatory) [18]. Thirty-seven dietary food components and products were used to calculate the DII score, including: 29 anti-inflammatory elements: monounsaturated fatty acids (MUFAs), PUFAs, n-3 fatty acids, n-6 fatty acids, fiber, alcohol, vitamins A, D, E, C, and B_6_, β-carotene, thiamine, riboflavin, niacin, folic acid, magnesium, selenium, zinc, flavan-3-ol, flavones, flavonols, flavonones, anthocyanidins, isoflavones, caffeine, garlic, onion, green/black tea, and 8 pro-inflammatory elements: carbohydrates, protein, total fat, SFAs, trans fat, cholesterol, iron, and vitamin B_12_. Energy-adjusted values (the nutrient density method) were used to decrease the influence of different energy intakes among study participants [35].

### 2.8. Statistical Analysis

Nominal variables are presented as n (% of group), continuous variables as mean ± SD or median (tercile [T]1; T3). Normality of distribution in subgroups was evaluated using Kolmogorov–Smirnov test, skewness, and kurtosis values, and based on visual assessment of histograms. Subgroups based on DII terciles were created. Comparison of parameters between DII tercile groups was made using chi-square test for nominal variables and with one-way ANOVA or Kruskal–Wallis test for continuous variables (as appropriate). Post-hoc test (Tukey test for ANOVA and Dunn test for Kruskal–Wallis test) was used with Bonferroni correction for multiple comparisons. Multivariate linear regression models were used adjusting for covariates: age, sex, place of living, marital status, education, smoking, alcohol, physical activity, and BMI. DII was included in regression models as both continuous and factorial variables (terciles). Additionally, we carried out a test for linear trend including the median value for each DII tercile as a continuous variable in the regression model and after adjusting for above mentioned covariates, which is a common approach in several studies from similar topics [36,37,38,39]. All tests were two-tailed with a significance level of 0.05. Statistical analysis was conducted using R software (A language and environment for statistical computing, version 3.5.1. R Foundation for Statistical Computing, Vienna, Austria).

The power analysis was performed in relation to one-way ANOVA with fixed effect and linear regression. Statistical significance level α = 0.05, N (sample size) = 1791, k (number of subgroups) = 3, equal size of subgroups (n = 597) was assumed for calculation. It was conducted using the software G*Power (version 3.1.9.6). In the ANOVA power test, the effect size f was used, which is defined as: f = σ_m_/σ, where σ_m_ is the standard deviation of the group mean µ_i_ and σ, the common standard deviation within k groups. The power analysis was calculated as 97%, which is much higher than 80%, considered commonly acceptable power level [40]. The effect size in the multiple regression is expressed as f^2^ = V_S_/V_E_, where V_S_ is the proportion of variance explained by a set of predictors, and V_E_ is the residual of error variance (V_E_ + V_S_ = 1). The proportion of variance explained is given by V_S_ = R^2^ and the residual variance by V_E_ = 1 − R^2^. The power analysis was calculated as 99.5%, which is much higher than 80%, considered commonly acceptable power level [41].

## 3. Results

The mean DII score of study participants was −0.15 ± 2.89, indicating slightly anti-inflammatory potential of their diets. The minimum DII score (anti-inflammatory) was −7.85, and the maximum DII score (pro-inflammatory) was 7.32 (unset data). Table 2 presents socioeconomic and lifestyle characteristics of 1791 study participants who were divided into terciles, according to the inflammatory potential of diets estimated by DII. Differences were reported in mean DII scores, gender, place of residence, education, smoking status, alcohol consumption, and physical activity level. The remaining data were not considered statistically significant.

Table 3 presents the nutritional value and food groups according to DII terciles. The average energy value, simple sugars, the proportion of energy from fats, SFAs, PUFAs, and cholesterol were significantly higher in T3 compared to T1. In contrast, the proportion of energy from protein, carbohydrates, and MUFAs was significantly higher in T1 compared to T3.

The average PUFA/SFA ratio was most favorable in T1 compared to T3. Diets of study participants in T1 had the highest content of fruits, vegetables, nuts, seeds, and raisins compared to T3, and the lowest content of juices, refined cereals, processed and unprocessed red meat, high-fat/processed poultry, sweets (total) and chocolate, fats except for vegetable oils, low-fat milk and dairy, high-fat cheese and cream, potatoes, French fries, and eggs. The content of unrefined cereals and low-fat poultry was similar in T1 and T2, and lower in T3. The content of mixed meals and potatoes was similar in T2 and T3. More fish was consumed by study participants in T3 compared to T1 and T2. Coffee consumption was higher in T1 compared to T2. The remaining components were considered not statistically significant.

Table 4 presents the relationships between participants’ diets and DII scores, taking into account confounding factors (age, sex, place of living, marital status, education, smoking status, alcohol, physical activity, and BMI). The energy value of the diets, the intake of simple sugars, the proportion of dietary energy from total fats, SFAs, PUFAs, and cholesterol level were reflected by positive DII scores. The proportion of dietary energy from protein, total carbohydrates, MUFAs, and the PUFA/SFA ratio was reflected by negative DII scores.

Pro-inflammatory diet, defined as T3, was negatively associated with the intake of fruits, vegetables, legumes, beverages, low-fat poultry, soups, nuts, seeds, raisins, and tea compared to their intake in T1. Participants in T3 consumed more fish, juices, refined cereals, processed and unprocessed red meat, high-fat/processed poultry, sweets (including chocolate), sugar and honey, fats except for vegetable oils, low-fat milk and dairy, high-fat cheese and cream, potatoes, French fries, alcohol, and eggs.

Table 5 compares DII scores with anthropometric, biochemical, and atherogenic risk factors for CVD. In the group of women, significantly higher WC and WHR were associated with more pro-inflammatory diets. TG concentrations were lower in T1 compared to T2 and T3. FG levels were significantly lower in T1 and T2 compared to T3. The mean value of AIP in all study participants indicated an increased risk of developing CVD regardless of the DII tercile. CRI was optimal in T1 group. Higher values of both indices were reported in T2 and T3 compared to T1.

## 4. Discussion

We evaluated the DII score to determine anti- and proinflammatory potential of a diet (its energy value, proportion of energy from carbohydrates, proteins and fats, content of dietary fiber, and cholesterol) in the context of CVD risk among residents of Lower Silesia. According to the 2021 European Society of Cardiology (ESC) [4] guidelines on CVD prevention, replacing SFAs with unsaturated fatty acids is associated with a reduced risk of coronary heart disease (CHD) [42,43,44]. Long-chain fatty acids found in vegetable oils, i.e., palm oil (palmitic acid, C16:0), and in meat and dairy (palmitic acid and stearic acid, C18:0), have been reported to activate a number of inflammatory pathways, including mitogen-activated protein kinase (MAPK), high activation of the nuclear factor-kB (NF-kB), and activator protein (AP)-1, which directly increases expression of toll-like receptors (TLRs), leading toward an increased local and peripheral inflammation [10].

We reported a higher proportion of energy intake from SFAs in most pro-inflammatory diets (β = 4.09 CI_95_ [3.71; 4.47], *p* < 0.001) which amounted to 14.47%. Similar results were reported by other authors using the DII score calculation [16,45,46,47]. A higher proportion of SFAs in pro-inflammatory diets (T3) can be related to the higher content of non-vegetable fats (β = 18.47 CI_95_ [18.63; 20.10], *p* < 0.001), which contribute to a higher risk of CHD [4]. In addition, the PUFA/SFA ratio was the least favorable in T3, i.e., 0.36 ± 0.12/1, and its higher values were negatively correlated with pro-inflammatory diet (β = −0.18 CI_95_ [−0.19; 0.16], *p* < 0.001) compared to DII in T1. The optimal PUFA/SFA ratio should be above 1.0 [28,29]. Lower levels of SFAs in the diet are associated with decreased consumption of foods high in dietary cholesterol whose higher intake increased with the inflammatory potential of the diet (T3 vs. T1 β = 144.62 CI_95_ [131.53; 157.72], *p* < 0.001). However, current guidelines no longer recommend an upper limit for dietary cholesterol intake, but rather focus on adopting healthy dietary patterns (e.g., the Mediterranean-style and DASH diets) which are inherently low in cholesterol [48]. In other studies based on DII score calculation, higher cholesterol intake was associated with a pro-inflammatory diet [45,46,47]. Similarly, dietary intake of eggs should be estimated according to the dietary pattern, because observations on egg consumption may be associated with other dietary components. Eggs can be consumed as part of an unhealthy dietary pattern, such as the Western pattern diet. In this study, eggs were associated with an overall unhealthy diet (T1 compared to T2 β = 6.29 CI_95_ [−4.52; 8.08], *p* < 0.001), similarly in other studies [49,50].

It is recommended to limit intake of added sugars to less than 10% of the daily total energy intake [51,52]. Higher intake of added sugars is associated with poorer diet quality, because consuming energy-dense foods low in nutrients leads to overweight and obesity that, in turn, increase the risk of type 2 diabetes and CVD [52]. Besides, higher intake of fructose causes dysbiosis of the microbiota, leading to an increased permeability of the gut barrier [53]. In this study, the intake of simple sugars was higher in T3 compared to T1 (β = 31.29 CI_95_ [26.56; 36.02], *p* < 0.001). The Mean daily intake of foods containing simple sugars was positively associated with T3 compared to T1, respectively: sweets except for chocolate (β = 26.01 CI_95_ [22.74; 29.29]; *p* < 0.001), juices (β = 25.83 CI_95_ [9.21; 42.54], *p* = 0.002), sugar and honey (β = 10.03 CI_95_ [8.17; 11.89], *p* < 0.001), and chocolate (β = 7.96 CI_95_ [6.39; 9.53], *p* < 0.001), which was consistent with the results of other studies using the DII score calculation [38,54].

Fruits, vegetables, and whole grain products are particularly recommended due to their high nutritional value. They are rich dietary sources of carotenoids, vitamin C, flavonoids, fiber, potassium, and magnesium [7,8]. Their higher intake is associated with lower serum CRP levels and a lower risk of elevating other pro-inflammatory markers [10]. In our study using the DII score calculation, participants’ diets indicated as more pro-inflammatory (T3) were negatively associated with fruits and vegetables intake compared to diets indicated as more anti-inflammatory (T1) (β = −55.41 CI_95_ [−77.85; −32.97], *p* < 0.001, β = −67.78 CI_95_ [−88.44; −47.12], *p* < 0.001, respectively), which is consistent with other studies using DII calculations [16,38,45,47,54]. Potatoes and French fries, which were not classified as vegetables, were positively associated with a pro-inflammatory diet (T3 vs. T1, respectively: β = 16.19 CI_95_ [9.18; 23.22], *p* < 0.001; β = 3.46 CI_95_ [2.45; 4.47], *p* < 0.001). Potatoes, rich in amylopectin starch, have a high glycemic index (GI) and load. The American Heart Association (AHA) guidelines state that dietary patterns rich in fruits and vegetables, with the exception for potatoes, are associated with a reduced risk of CVD [5].

Similarly, refined grains were positively associated with T3 compared to T1 (β = 57.28 CI_95_ [50.39;65.17], *p* < 0.001). Refined grains contain less dietary fiber, vitamins and minerals than their whole grain counterparts, have a higher GI, and may increase inflammation [10,38,50].

In addition, a pro-inflammatory diet was positively associated with processed red/mixed meat (β = 22.75 CI_95_ [19.12; 26.38], *p* < 0.001), high fat/processed poultry (β = 16.17 CI_95_ [12.47; 19.87], *p* < 0.001, respectively), and red meat (β = 7.57 CI_95_ [5.59; 9.54], *p* < 0.001), which is consistent with other studies using DII calculations [38,45,47,50]. According to the 2021 ESC guidelines on CVD prevention, consumption of processed and unprocessed meat was associated with a higher risk of atherosclerotic cardiovascular disease (ASCVD) by, respectively, 7% and 3% [52]. Besides, their lower consumption is additionally beneficial due to salt intake reduction [4]. In this study, among all types of meat products, the pro-inflammatory diet (T3) was negatively associated with low-fat poultry, a recommended protein source in healthy dietary patterns (T3 compared to T1 β = −1.99 CI_95_ [−3.66; −0.63], *p* = 0.004) [55].

The 2021 AHA [5] dietary guidance to improve cardiovascular health recommends choosing mostly protein from plants (legumes and nuts). In our study, mean intake of legumes and nuts was negatively associated with the pro-inflammatory diet compared to the anti-inflammatory diet (respectively: β = −2.37 CI_95_ [−4.41; 0.34], *p* = 0.023 and β = −2.55 CI_95_ [−4.92; −0.17], *p* = 0.034), similarly in other studies [16,45,50]. Most legumes contain phytochemicals: bioactive compounds, including enzyme inhibitors, phytohemagglutinins (lectins), phytoestrogens, oligosaccharides, saponins, and phenolic compounds, which may provide health benefits, protecting against diseases or disorders such as CVD and inflammation [56]. The most abundant and active isoflavone in soy is genistein, which acts as a natural selective estrogen receptors-β modulator and positively regulates some cardiovascular risk markers [57]. Squadrito et al. [58] in a randomized trial, including 120 postmenopausal women with metabolic syndrome (MetS), found that one year of treatment with genistein improved surrogate endpoints associated with risk for diabetes and CVD. Among three prospective cohorts of US men and women, a higher intake of isoflavones and tofu was associated with a moderately lower risk of developing CHD (isoflavones: pooled hazard ratio [HR] comparing the extreme quintiles: 0.87 [95%CI, 0.81–0.94]; *p* = 0.008, tofu: pooled HRs [95%CIs] of 0.82 [0.70–0.95; *p* = 0.005]). In addition, among women the favorable association of tofu was more pronounced in young women and postmenopausal women without hormone therapy (P_interaction_ = 0.002) [59].

In our study, dairy products, regardless of fat content, were positively associated with pro-inflammatory diet. Park et al. [60] investigated the associations between dairy product intake and hypertriglyceridemia in obese Korean adults, but a recent systematic review [61] did not confirm any association between consumption of dairy products and a pro-inflammatory effect in healthy individuals, or the association of low- and regular-fat dairy consumption with higher risk of CVD, except for a positive association of high-fat milk and an inverse association of cheese with CHD risk. In Spanish [16] and Mexican [50] studies higher dairy intake was associated with anti-inflammatory diets, but the results of the Italian study [45] were similar to our own.

Authors of epidemiological studies indicate decreased risk of CVD among abstainers and that any amount of alcohol increases blood pressure and BMI [4]. In our study, alcohol consumption was positively associated with a pro-inflammatory diet compared to an anti-inflammatory diet (T3 compared to T1 β = 22.06 CI_95_ [7.49;36.64], *p* = 0.003). In the TOSCA.IT study, higher alcohol consumption was associated with a pro-inflammatory diet (Q4) compared to an anti-inflammatory diet (Q1), *p* < 0.0001 [45]. However, an inverse association was observed in the Diabetes Mellitus Survey administered in Mexico City (DMS-MC), where alcohol intake in Q5 (pro-inflammatory diet) was lower than in Q1 (anti-inflammatory diet, *p* < 0.0001) [50].

In our study, higher intake of coffee was assessed in DII T1 compared to DII T2 and DII T3, but after excluding confounding factors, only tea was negatively associated with DII T3 compared to DII T1. Contrasting results were obtained in the Korean study, where coffee and tea intake was reduced in study participants with more pro-inflammatory diets [46]. Phenolic compounds found in coffee and tea have anti-inflammatory and antioxidant effects. In addition to a reduction of pro-inflammatory markers (IL-1, IL-6, and TNF-α), phenolic compounds also lower LDL-C oxidation, leading to decreased vascular inflammation, risk of platelet aggregation, and a reduction in oxidative stress and nitric oxide (NO) effects [8]. However, according to the ESC 2021 guidelines, unfiltered coffee should be consumed in moderation due to its lipid-raising components: kahweol and cafestol [62].

Our study raises some contentious issues regarding fish and soft drinks. According to the ESC 2021 guidelines, it is recommended to avoid sweetened beverages, including fruit juices, as well as sweetened carbonated and non-carbonated soft drinks [4,5]. In the European Prospective Investigation into Cancer and Nutrition (EPIC) study, consumption of artificially sweetened and sugar-sweetened soft drinks was associated with overall mortality, and consumption of artificially sweetened soft drinks was directly associated with CVD [51]. In our study, after excluding confounding factors, soft drinks were negatively associated with a pro-inflammatory diet. In other studies based on DII scores, higher consumption of soft drinks was associated with a more pro-inflammatory diet [38,47,50].

Oily fish should be consumed twice a week as a source of omega-3 fatty acids. However, in contrast to other analyses based on DII score calculations [16,38,45,50,54], in this study overall mean fish consumption was higher in participants with a pro-inflammatory diet (T3 compared to T1 β = 1.41 CI_95_ [0.09; 2.72], *p* = 0.035). This can be associated with the fact that half of the consumed fish (53.9%, unset data) was lean (i.e., cod) and processed (coated with batter and breading). The authors of another dietary inflammatory index determining anti-inflammatory potential of diets classified fish as pro-inflammatory due to inappropriate preparation methods [63]. A recent meta-analysis involving individual data of 191,558 people from 58 countries found that eating 175 g of fish per week was associated with a significant reduction in the risk of CVD events (16%) and decreased death rate (18%) in secondary prevention. The benefits were observed only for oily fish, preferably not fried [64]. Well-done or browned fried fish may have a stronger pro-inflammatory potential and increase the risk of chronic diseases [65].

The Western pattern diet is characterized by a higher intake of proinflammatory (T3) food products, i.e., refined grains, simple sugars, red and processed meat, eggs, high-fat dairy, and low intake of fruits, vegetables, whole grains, nuts, or legumes [12]. This diet contributes to weight gain and to the proliferation of visceral adipose tissue which, as an endocrine organ significantly contributes to inflammatory processes by releasing pro-inflammatory factors, including leptin, TNF- α, and IL-6 [10]. Besides, such diet is characterized by a higher content of pro-inflammatory advanced glycation end-products (AGE’s). However, it is worth noting that the method of cooking (i.e., frying) has a significant impact on AGE formation. Chronic low-grade systemic inflammation and a pro-inflammatory diet may increase CVD risk and severity [8]. Plant-based (PB) diets are associated with good health and are also recommended for environmental sustainability. The Mediterranean diet has also been included in definitions of PB, due to the emphasis on some components [66]. Kent et al. [66] found that participants on the PB diet more often met recommended intakes of carbohydrates, dietary fiber, and vitamin E, and less often met recommendations for protein, vitamin B_12_, and iodine compared to omnivores. Intakes of protein, omega-3 fatty acids, iron, and zinc were sufficient from the PB diet. It is worth emphasizing that the bioavailability of these nutrients is lower in PB diet compared to animal-derived products [66]. Recent high-quality evidence supports the Mediterranean diet (rich in i.e., vegetables, fruits, wholegrains, legumes, nuts, and olive oil) in secondary prevention of CVD with impacts on atherosclerosis progression. It may be caused by the reduction of systemic inflammation, irrespective of changes in weight or cholesterol. The Mediterranean diet is characterized by a low DII, showing its anti-inflammatory potential [67]. A healthy balanced diet, adjusted energy intake, and expenditure to achieve and maintain a healthy body weight with proper supplementation could provide a possible further strategy to effectively prevent and control noncommunicable diseases. An et al. [68], in the 884 randomized controlled intervention trials evaluating 27 types of micronutrients among 883,627 participants, found that omega-3 fatty acids supplementation decreased CVD mortality (relative risk [RR]: 0.93; 95%CI: 0.88–0.97), myocardial infarction (RR: 0.85; 95%CI: 0.78–0.92), and CHD events (RR: 0.86; 95%CI: 0.80–0.93). Folic acid supplementation decreased the stroke risk (RR: 0.84; 95%CI: 0.72–0.97), and coenzyme Q10 supplementation decreased all-cause mortality events (RR: 0.68; 95%CI: 0.49–0.94). Additionally, Pontes et al. [69], based on twenty-six randomized controlled trials (n = 1720) found a significant effect of probiotics in reducing body weight (mean deviation [MD]:−0.70 kg; 95%CI:−1.04, −0.35 kg; *p <* 0.0001), BMI (MD:−0.24 kg/m^2^; 95%CI:−0.35, −0.12 kg/m^2^; *p* = 0.0001), WC (MD:−1.13 cm; 95%CI:−1.54, −0.73 cm; *p <* 0.0001), fat mass (MD:−0.71 kg; 95%CI:−1.10, −0.32 kg; *p* = 0.0004), TNF-α (MD:−0.16 pg/mL; 95%CI:−0.24, −0.08 pg/mL; *p* = 0.0001), insulin (MD:−0.85 mcU/mL; 95%CI:−1.50, −0.21 mcU/mL; *p* = 0.010), TC (MD:−0.16 mmol/L; 95%CI:−0.26, −0.05 mmol/L; *p* = 0.003), and LDL-C (MD:−0.09 mmol/L; 95%CI:−0.16, −0.03 mmol/L; *p* = 0.006) compared with control groups. They observed a substantial decrease in body weight, BMI, and WC using both single and multi-bacterial species.

In our study we also evaluated the association of the DII score with CVD risk parameters among urban and rural residents of Lower Silesia (Table 5). The anti-inflammatory diet, according on DII score calculations, was associated with lower WHR and WC in the group of women and lower TG, FG, and atherogenicity indices in the group of men and women, confirming the benefits of an anti-inflammatory diet on CVD-related parameters. In another Polish study, WC was associated with a pro-inflammatory diet, but only in the group of men [70]. Other study has also indicated an association between WC and WHR with pro-inflammatory values on the DII [16].

Obesity is a low-grade chronic inflammation due to an imbalance between intake and expenditure of energy. Similar to our study results, other authors have also found an association between a pro-inflammatory diet and higher dietary energy expenditure based on the DII score calculation [47,71]. The storage of excess energy in adipocytes results in hyperplasia and hypertrophy adipose tissue, associated with the release of macrophages secreting high levels of pro-inflammatory receptors toll-like receptors (TLRs), tumor necrosis factor receptors (TNFRs), interleukin−1- receptor (IL-1R), and activation of NF-kB transcription factors for pro-inflammatory molecules. As a further consequence, low-grade inflammation can affect insulin sensitivity leading to impaired metabolism and an increased risk of other non-communicable diseases [72,73]. In addition, excess lipids are redirected into other organs (liver, skeletal muscle, and blood vessels), inducing the expression of pro-inflammatory mediators, differentiation of monocytes into macrophages, and M1 systemic macrophages recruitment. This may lead to a vicious cycle characterized by increased central fat, intrahepatic fat accumulation, vascular inflammation, and impaired endothelial function [74].

In our study, participants with DII T3 diets consumed more dietary energy from fats. The consequences of an excessive fat intake high-fat diet (HFD), besides obesity, hyperinsulinemia, dyslipidemia, comprise dysbiosis, gut barrier dysfunction, and increased intestinal permeability, can strongly contribute to the development of low-grade systemic inflammation [12]. The microbiome of the inhabitants of the Lower Silesia region is worth assessing in future studies in order to better formulate dietary recommendations.

The Geelong Osteoporosis Study (GOS) involving 1363 men, found that the adjusted odds ratio (OR, 95%CI) for CVD risk factors was 2.0 (1.01–3.96) for individuals with pro-inflammatory diet compared to individuals with anti-inflammatory diet, as indicated by higher DII scores [75]. Similarly, authors of the Primary Prevention of Cardiovascular Disease with a Mediterranean Diet (PREDIMED) study, which included 7216 participants (men aged 55–80 years and women aged 60–80 years) with high CVD risk, after medial follow-up of 4.8 years, diagnosed CVD in 277 study participants consuming a pro-inflammatory diet. The adjusted HR (95%CI) for CVD in Q4 vs. Q1 was 1.73 (1.15–2.60) [15]. In the SUN study, the HR for participants between the highest (Q1) and the lowest quartile (Q5) was 2.03 (95%CI; 1.06–3.88), proving a linear trend with overall CVD risk [76]. In contrast, the SU.VI.MAX study, which included 7743 men and women (11.4-year follow-up), found no statistically significant association between DII score and the risk of cardio-metabolic disorders (CMDs) [17].

To the best of our knowledge, our study is the first to assess atherogenic indices depending on DII scores and accurately determine different product groups in DII terciles, while considering demographic confounding factors. Recent studies have investigated the association between a pro-inflammatory diet, as determined by DII score, and the increased risk of dyslipidemia [71], elevated triglycerides/HDL-C, and apolipoprotein (B) [77]. A Brazilian study [78] supported the hypothesis that a pro-inflammatory diet is associated with a higher atherogenic risk in schoolchildren. Determination of atherogenicity indices is a noteworthy method to complement cardiometabolic risk screening and monitoring [27,79,80].

Due to the cross-sectional nature of the study, it was impossible to establish the association between DII scores and selected CVD risk factors. However, this study design allowed for the assessment of the relationship between the variables and establish management strategies to protect health. Moreover, it is unclear whether overweight or obese individuals are more likely to choose pro-inflammatory diets, or whether pro-inflammatory diets contribute to promoting obesity. This should be confirmed in prospective analyses. The fact that this study was carried out with standardized methods and a validated high-quality FFQ including 154 food products and dishes specific to the Lower Silesia region, is a definite strength of the study. However, this method is limited, because some DII components were not included in the questionnaire (i.e., saffron, eugenol, ginger, turmeric, pepper, rosemary, and thyme). However, this is the first cross-sectional study to determine the inflammatory potential of the diets of Poland’s Lower Silesia inhabitants, in which DII scores were calculated based on 37 food components and products. Also, due to the cross-sectional nature of the study design, study results correspond to the actual dietary habits of study participants.

So far, no studies have identified product groups specific to each DII tercile after excluding confounding factors, as assessed in this study (Table 4). To conduct more thorough analysis/get more accurate results, future studies should assess the role of inflammatory markers. The results of this study are informative and provide an important basis for further research on the quality of diet and nutrition.

## 5. Conclusions

Among participants with pro-inflammatory diets, we reported higher mean values of TG, FG, API, and CRI in the group of men and women, and higher WC and WHR in the group of women. Study participants on pro-inflammatory diets consumed more refined grain products, sweets, juices, red meat, high-fat cheese and cream, alcohol, fats (except for vegetable oils), potatoes, sugar and honey, French fries, fried fish, and processed/high-fat poultry. Moreover, we reported higher consumption of milk, low-fat dairy, and eggs in study participants with pro-inflammatory diets, which may be due to the fact that these food products are associated with unhealthy dietary habits. However, their consumption should not be considered as an independent CVD risk factor. Anthropometric and biochemical parameters were more favorable among study participants whose diets had higher content of vegetables, fruits, nuts, seeds, raisins, pulses, low-fat poultry, and tea. However, the association of beverage consumption with dietary inflammatory potential requires further study.

## Figures and Tables

**Table 1:** Characteristics of the food groups.

No.	Food Groups	FFQ Dietary Products
1.	Milk and low-fat dairy	Low-fat milk, 1–2% fat, milk, 3.2% fat, buttermilk, 0.5% fat, cocoa with milk, cottage cheese, quark, fresh cheese, low-fat yoghurt, yogurt, 2–8% fat, kefir
2.	High-fat cheese and cream	Feta cheese, hard cheese, cheese, “fromage” naturel, cheese, Edam type, fat, cream, 12% fat, cream, 18% fat
3.	Fats without oils	Margarine, soft, butter, lard, Finea/Masmix, mayonnaise
4.	Fruits	Apple, banana, grapefruit, grapes, tangerine, strawberries, kiwi fruit, lemon, orange, peach, pear, plum, raspberries
5.	Vegetables	Beets, cooked, broccoli, green, cabbage, red (raw), cabbage, Shantung, cabbage, white (raw), cabbage, white (boiled), carrot (fresh), carrot (boiled), cauliflower (raw), cauliflower (boiled with butter), chives, cucumber (raw), garlic (raw), lettuce, mushroom (fried), onion (raw), parsley, leaves, horseradish, pepper (cooked), pepper, red (raw), radish, tomato (raw), tomato (cooked), tomato sauce, spinach (cooked), squash, summer (cooked), string beans (boiled),sweet corn (canned, drained), shantung cabbage, salad with mayonnaise, sauerkraut salad, lettuce with sour cream salad
6.	Legumes	Beans, white (boiled), peas green (canned, drained)
7.	Chips	Potato (French fried)
8.	Potatoes	Potato (boiled), potato (mashed)
9.	Red meat	Beef steaks, pork, belly (no bone, boiled), pork cutlets (breaded, fried), organ meat (liver, tongue, heart), beef and pork minced cutlets (fried)
10.	Processed red/mixed meat	Beef, ham (cooked), Frankfurter/Hotdog, luncheon meat (pork), pork ham sausage Slaska (pork, cooked), sausage Krakowska (pork and beef), sausage Biala (pork), sausage Szynkowa (turkey), Head Cheese, white and black chicken pate (canned)
11.	Low-fat poultry	Low-fat poultry Chicken without skin (cooked/fried), turkey (roasted)
12.	High-fat/processed poultry	Chicken fillets (breaded, fried), chicken ham, chicken with skin (cooked/fried), turkey, ham
13.	Fish	Cod fillets (breaded and fried), herring in cream, mackerel (smoked)
14.	Unrefined grains	Rye, brown bread, wheat-rye bread with sunflower seeds, pasta (cooked), buckwheat groats (boiled), pearl barley groats (boiled), soup, milk with rolled oats
15.	Refined grains	Wheat bread, rice (boiled), wheat rolls (kajzerki), wheat rolls (wroclawskie), wheat-rye bread/white bread, cold cereal (cornflakes)
16.	Mixed dishes	Baked beans with meat, cabbage leaves, stuffed, Polish dumplings, with meat, sauerkraut with sausage and meat (bigos, stewed), dumplings with potato filling (Ruskie, boiled), vegetable salad (cooked with mayonnaise)
17.	Soups	Broth, soup with vegetables, soup, Krupnik with pearl barley groats, soup, Zurek sour rye, soup, tomato, soup, sauerkraut, soup, white bean
18.	Juices	Orange juice, carrot juice, apple juice, grapefruit juice, blackcurrant juice, multifruit juice from Polish fruits, multifruit juice from exotic fruits
19.	Beverages	Raspberry juice, fruits drink, soft drink (regular), soft drink (low calorie)
20.	Alcohol	Beer, red wine, vodka
21.	Sweets	Ice cream, biscuits, yeast cake, short-cake, gingerbread cake, sponge cake, cheesecake (Krakowski), halva with vanilla, drops, sweets
22.	Chocolate	Bitter chocolate, milk chocolate
23.	Sugar and honey	Honey, sugar
24.	Nuts, seeds and raisins	Nuts, raisins, dried, seeds, walnuts
25.	Eggs	Eggs boiled/fried
26.	Coffee	Coffee
27.	Tea	Tea, green/herb, Tea, black

**Table 2:** Characteristics of 1791 participants of PURE Poland study population by dietary inflammatory index (DII) terciles.

	Total Group n = 1791	Tercile 1 n = 597	Tercile 2 n = 597	Tercile 3 n = 597	*p*
DII, mean ± SD	−0.15 ± 2.89	−3.37 ± 1.44	−0.15 ± 0.77	3.06 ± 1.34	<0.001
Sex, n (%)					
Female	1120 (62.5)	446 (74.7)	372 (62.3)	302 (50.6)	<0.001
Male	671 (37.5)	151 (25.3)	225 (37.7)	295 (49.4)	
Age, years, mean ± SD	54.61 ± 9.80	54.87 ± 8.63	54.35 ± 9.38	54.62 ± 11.21	0.656
Place of living, n (%)					
Rural	699 (39.0)	115 (19.3)	236 (39.5)	348 (58.3)	<0.001
Urban	1092 (61.0)	482 (80.7)	361 (60.5)	249 (41.7)	
Marital status, n (%)					
Married/living together	1334 (74.5)	435 (72.9)	449 (75.3)	450 (75.4)	0.290
Never married	129 (7.2)	54 (9.0)	41 (6.9)	34 (5.7)	
Separated/divorced/widowed	327 (18.3)	108 (18.1)	106 (17.8)	113 (18.9)	
Education, n (%)					
Primary/trade	538 (30.0)	96 (16.1)	170 (28.5)	272 (45.6)	<0.001
Secondary and high secondary	703 (39.3)	234 (39.2)	263 (44.1)	206 (34.5)	
University	550 (30.7)	267 (44.7)	164 (27.5)	119 (19.9)	
Smoking, n (%)					
Currently Uses Tobacco Products	372 (20.8)	108 (18.1)	121 (20.3)	143 (24.0)	0.029
Formerly Used Tobacco Products	570 (31.8)	210 (35.2)	195 (32.7)	165 (27.6)	
Never Used Tobacco Products	849 (47.4)	279 (46.7)	281 (47.1)	289 (48.4)	
Alcohol, n (%)					
Currently use alcohol products	1237 (69.1)	426 (71.4)	404 (67.7)	407 (68.2)	0.001
Formerly used alcohol products	177 (9.9)	44 (7.4)	51 (8.5)	82 (13.7)	
Never used alcohol products	377 (21.0)	127 (21.3)	142 (23.8)	108 (18.1)	
Physical activity, n (%)					
Low and moderate	505 (28.2)	202 (33.8)	170 (28.5)	133 (22.3)	<0.001
High	1286 (71.8)	395 (66.2)	427 (71.5)	464 (77.7)	

Tercile groups compared with chi-square test for nominal variables and with ANOVA analysis for age.

**Table 3:** Nutrients intake and food groups according to the terciles of the dietary inflammatory index (DII) among 1791 participants of PURE Poland study population.

Parameter	Total Group	Tercile 1	Tercile 2	Tercile 3	*p*	Post-Hoc
**Nutrients intake**						
Energy (kcal/day)	2032.03 ± 657.34	1660.31 ± 529.41	2011.53 ± 614.86	2424.26 ± 588.61	<0.001	1 < 2 < 3
Fiber (g/day)	30.72 ± 11.52	30.61 ± 11.92	31.13 ± 12.37	30.41 ± 10.16	0.542	
Sugars (g/day)	91.58 ± 40.27	77.00 ± 32.90	89.68 ± 38.54	108.08 ± 42.59	<0.001	1 < 2 < 3
Protein intake (% energy)	15.08 ± 2.12	15.55 ± 2.17	15.08 ± 2.11	14.61 ± 1.97	<0.001	1 > 2 > 3
Total fat intake (% energy)	31.99 ± 5.75	29.41 ± 5.09	31.63 ± 5.29	34.94 ± 5.46	<0.001	1 < 2 < 3
Carbohydrates intake (% energy)	54.32 ± 6.77	57.12 ± 6.72	54.61 ± 6.24	51.22 ± 5.98	<0.001	1 > 2 > 3
SFA (% energy)	12.37 ± 3.48	10.61 ± 2.58	12.02 ± 3.15	14.47 ± 3.49	<0.001	1 < 2 < 3
PUFA (% energy)	10.87 ± 2.13	10.11 ± 2.13	10.83 ± 2.00	11.69 ± 1.96	<0.001	1 < 2 < 3
MUFA (% energy)	4.99 ± 1.04	5.11 ± 1.16	5.02 ± 1.01	4.83 ± 0.92	<0.001	1 > 2, 3
PUFA/SFA	0.44 ± 0.15	0.51 ± 0.16	0.44 ± 0.13	0.36 ± 0.12	<0.001	1 > 2 > 3
Cholesterol (mg/day)	277.62 ± 128.53	203.33 ± 86.87	260.44 ± 97.41	369.10 ± 135.54	<0.001	1 < 2 < 3
**Food groups**						
Fruits (g/day)	293.44 ± 184.37	332.84 ± 201.11	297.71 ± 185.60	249.79 ± 154.02	<0.001	1 > 2 > 3
Vegetables (g/day)	281.40 ± 175.17	340.01 ± 206.55	273.11 ± 172.99	231.10 ± 116.29	<0.001	1 > 2 > 3
Legumes (g/day)	17.38 (5.57;17.38)	12.14 (5.57;17.38)	17.38 (5.57;17.38)	17.38 (5.57;23.95)	0.154	
Fish (g/day)	13.11 (6.56;17.56)	9.84 (6.56;16.98)	13.11 (6.56;17.56)	13.70 (9.84;20.84)	<0.001	1, 2 < 3
Beverages (g/day)	49.18 (16.39;250.00)	35.71 (0.00;266.39)	49.18 (16.39;212.82)	52.11 (16.39;212.82)	0.436	
Juices (g/day)	101.29 (32.79;178.57)	81.97 (16.39;139.93)	101.29 (35.71;172.72)	114.75 (49.18;214.29)	<0.001	1 < 2 < 3
Refined grains (g/day)	75.57 ± 62.94	41.53 ± 46.20	73.79 ± 57.32	111.39 ± 63.49	<0.001	1 < 2 < 3
Unrefined grains (g/day)	63.96 ± 47.54	65.64 ± 46.07	67.13 ± 51.99	59.12 ± 43.89	0.008	1, 2 > 3
Red meat (g/day)	25.95 ± 16.63	20.64 ± 14.40	26.92 ± 17.15	30.30 ± 16.76	<0.001	1 < 2 < 3
Processed red/mixed meat (g/day)	46.04 ± 33.16	31.24 ± 23.75	43.99 ± 29.88	62.89 ± 36.58	<0.001	1 < 2 < 3
Low-fat poultry (g/day)	6.56 (0.00;14.29)	8.52 (1.97;14.29)	8.52 (1.97;14.29)	6.56 (0.00;14.29)	<0.001	1, 2 > 3
High-fat/processed poultry (g/day)	43.11 ± 31.39	33.43 ± 25.90	42.03 ± 30.73	53.88 ± 33.66	<0.001	1 < 2 < 3
Mixed dishes (g/day)	32.79 (19.67;40.52)	26.23 (13.11;39.34)	32.79 (19.67;40.52)	32.79 (20.84;41.69)	<0.001	1 < 2, 3
Soups (g/day)	244.50 ± 138.77	241.87 ± 156.71	253.30 ± 142.95	238.33 ± 112.77	0.150	
Sweets without chocolate (g/day)	38.04 ± 28.82	25.09 ± 20.40	36.37 ± 26.39	52.67 ± 31.57	<0.001	1 < 2 < 3
Chocolate (g/day)	6.56 (3.28;7.14)	3.28 (0.00;7.14)	6.56 (3.28;7.14)	6.56 (3.28;10.42)	<0.001	1 < 2 < 3
Sugar and honey (g/day)	16.64 ± 15.78	10.52 ± 12.30	16.96 ± 15.47	22.46 ± 16.90	<0.001	1 < 2 < 3
Fats without oils (g/day)	19.81 ± 15.75	11.18 ± 7.94	17.31 ± 12.31	30.94 ± 18.04	<0.001	1 < 2 < 3
Milk and low-fat dairy	152.14 (72.83;290.46)	117.50 (57.75;238.21)	147.86 (65.74;285.71)	190.27 (98.14;339.15)	<0.001	1 < 2 < 3
High-fat cheese and cream	33.97 ± 24.25	24.19 ± 19.39	33.36 ± 23.70	44.36 ± 24.98	<0.001	1 < 2 < 3
Potatoes	82.13 ± 57.09	72.09 ± 54.85	86.40 ± 57.60	87.90 ± 57.52	<0.001	1 < 2, 3
Chips	0.00 (0.00;7.54)	0.00 (0.00;7.54)	0.00 (0.00;7.54)	7.54 (0.00;16.43)	<0.001	1 < 2 < 3
Nuts, seeds and raisins	10.36 (1.43;14.69)	11.02 (5.44;18.20)	10.36 (2.57;14.80)	6.06 (0.00;11.24)	<0.001	1 > 2 > 3
Alcohol	12.13 (0.00;49.67)	12.13 (0.00;47.14)	12.13 (0.00;47.14)	15.45 (0.00;56.45)	0.436	
Eggs	19.29 (6.43;19.29)	6.43 (2.95;19.29)	6.43 (6.43;19.29)	19.29 (6.43;19.29)	<0.001	1 < 2 < 3
Coffee	326.77 ± 264.55	344.47 ± 270.43	303.35 ± 251.89	332.47 ± 269.65	0.022	1 > 2
Tea	427.18 ± 321.04	429.33 ± 344.07	431.27 ± 323.93	420.93 ± 293.56	0.840	

Data presented as mean ± SD or median (tercile [T1];T3), depending on data distribution. Tercile groups compared with ANOVA analysis or Kruskal–Wallis test. For ANOVA-post-hoc Tukey test was applied, for Kruskal–Wallis test-post-hoc Dunn test was applied. PUFA: Polyunsaturated fatty acid, MUFA: Monounsaturated fatty acid, and SFA: Saturated fatty acid. To calculate DII scores, energy-adjusted values (the nutrient density method) were used to decrease the influence of different energy intakes among study participants.

**Table 4:** Associations between dietary inflammatory index (DII) and diet ingredients in total study group.

Parameter	DII Continuous ^a,d^			DII Terciles ^b,d^			*p*-Trend ^c,d^
	β	95%CI for β	*p*	T1	T2: β (95%CI)	T3: β (95%CI)	
**Nutrients intake**							
Energy (kcal/day)	0.002	0.0018 to 0.0022	<0.001	Ref.	328.93 (261.65 to 396.22)	729.40 (657.38 to 801.41)	<0.001
Fiber (g/day)	0.01	−0.003 to 0.018	0.183	Ref.	0.81 (−0.52 to 2.15)	0.71 (−0.72 to 2.14)	0.336
Sugars (g/day)	0.02	0.019 to 0.025	<0.001	Ref.	12.83 (8.41 to 17.25)	31.29 (26.56 to 36.02)	<0.001
Protein intake (% energy)	−0.21	−0.27 to −0.15	<0.001	Ref.	−0.41 (−0.66 to −0.17)	−0.86 (−1.12 to −0.60)	<0.001
Total fat intake (% energy)	0.19	0.17 to 0.21	<0.001	Ref.	2.09 (1.49 to 2.71)	5.29 (4.63 to 5.94)	<0.001
Carbohydrates intake (% energy)	−0.14	−0.16 to −0.12	<0.001	Ref.	−2.31 (−3.08 to −1.58)	−5.42 (−6.20 to −4.64)	<0.001
SFA (% energy)	0.39	0.36 to 0.42	<0.001	Ref.	1.53 (1.17 to 1.88)	4.09 (3.71 to 4.47)	<0.001
PUFA (% energy)	0.33	0.27 to 0.39	<0.001	Ref.	0.48 (0.25 to 0.71)	1.12 (0.87 to 1.36)	<0.001
MUFA (% energy)	−0.46	−0.57 to −0.34	<0.001	Ref.	−0.19 (−0.31 to −0.07)	−0.48 (−0.60 to −0.35)	<0.001
PUFA/SFA	−8.27	−8.96 to −7.58	<0.001	Ref.	−0.08 (−0.09 to −0.06)	−0.18 (−0.19 to −0.16)	<0.001
Cholesterol (mg/day)	0.01	0.011 to 0.013	<0.001	Ref.	46.68 (34.44 to 58.91)	144.62 (131.53 to 157.72)	<0.001
**Food groups**							
Fruits (g/day)	−0.002	−0.002 to −0.001	<0.001	Ref.	−22.80 (−43.77 to −1.83)	−55.41 (−77.85 to −32.97)	<0.001
Vegetables (g/day)	−0.003	−0.003 to −0.002	<0.001	Ref.	−46.47 (−65.77 to −27.16)	−67.78 (−88.44 to −47.12)	<0.001
Legumes (g/day)	−0.007	−0.01 to 0.001	0.082	Ref.	−1.52 (−3.43 to 0.38)	−2.37 (−4.41 to −0.34)	0.023
Fish (g/day)	0.01	0.0003 to 0.02	0.045	Ref.	−0.25 (−1.48 to 0.98)	1.41 (0.09 to 2.72)	0.035
Beverages (g/day)	−0.001	−0.0007 to 0.001	0.084	Ref.	−47.96 (−88.17 to −7.76)	−44.49 (−87.52 to −1.45)	0.045
Juices (g/day)	0.002	0.001 to 0.002	0.001	Ref.	13.84 (−1.69 to 29.37)	25.83 (9.21 to 42.45)	0.002
Refined grains (g/day)	0.017	0.015 to 0.019	<0.001	Ref.	25.51 (19.07 to 31.94)	57.28 (50.39 to 65.17)	<0.001
Unrefined grains (g/day)	−0.001	−0.003 to 0.002	0.582	Ref.	4.19 (−1.34 to 9.71)	−0.87 (−6.78 to 5.05)	0.759
Red meat (g/day)	0.03	0.02 to 0.04	<0.001	Ref.	4.89 (3.05 to 6.73)	7.57 (5.59 to 9.54)	<0.001
Processed red/mixed meat (g/day)	0.03	0.02 to 0.03	<0.001	Ref.	8.37 (4.98 to 11.77)	22.75 (19.12 to 26.38)	<0.001
Low-fat poultry (g/day)	−0.02	−0.03 to −0.01	0.001	Ref.	−0.16 (−1.44 to 1.11)	−1.99 (−3.36 to −0.63)	0.004
High-fat/processed poultry (g/day)	0.02	0.017 to 0.024	<0.001	Ref.	6.44 (2.98 to 9.90)	16.17 (12.47 to 19.87)	<0.001
Mixed dishes (g/day)	0.01	0.001 to 0.01	0.024	Ref.	3.24 (0.82 to 5.66)	2.09 (−0.50 to 4.68)	0.119
Soups (g/day)	−0.001	−0.002 to 0.001	0.071	Ref.	2.59 (−13.58 to 18.75)	−19.62 (−36.93 to −2.32)	0.025
Sweets without chocolate (g/day)	0.04	0.03 to 0.04	<0.001	Ref.	10.57 (7.51 to 13.63)	26.01 (22.74 to 29.29)	<0.001
Chocolate (g/day)	0.05	0.04 to 0.06	<0.001	Ref.	4.55 (3.08 to 6.02)	7.96 (6.39 to 9.53)	<0.001
Sugar and honey (g/day)	0.05	0.04 to 0.05	<0.001	Ref.	5.39 (3.66 to 7.14)	10.03 (8.17 to 11.89)	<0.001
Fats without oils (g/day)	0.09	0.09 to 0.10	<0.001	Ref.	5.27 (3.74 to 6.79)	18.47 (16.83 to 20.10)	<0.001
Milk and low-fat dairy (g/day)	0.003	0.003 to 0.004	<0.001	Ref.	48.32 (24.79 to 71.84)	120.85 (95.68 to 146.03)	<0.001
High-fat cheese and cream (g/day)	0.04	0.03 to 0.05	<0.001	Ref.	10.55 (7.93 to 13.18)	22.58 (19.77 to 25.39)	<0.001
Potatoes (g/day)	0.005	0.003 to 0.007	<0.001	Ref.	13.22 (6.66 to 19.79)	16.19 (9.18 to 23.22)	<0.001
Chips (g/day)	0.06	0.04 to 0.07	<0.001	Ref.	1.13 (0.18 to 2.08)	3.46 (2.45 to 4.47)	<0.001
Nuts, seeds and raisins (g/day)	−0.006	−0.01 to 0.01	0.075	Ref.	0.65 (−1.56 to 2.87)	−2.55 (−4.92 to −0.17)	0.034
Alcohol (g/day)	0.002	0.001 to 0.003	0.002	Ref.	10.70 (−2.91 to 24.32)	22.06 (7.49 to 36.64)	0.003
Eggs (g/day)	0.04	0.03 to 0.04	<0.001	Ref.	1.04 (−0.62 to 2.70)	6.29 (4.52 to 8.08)	<0.001
Coffee (g/day)	−0.003	−0.01 to 0.001	0.895	Ref.	−37.19 (−66.59 to −7.80)	−4.94 (−36.41 to 26.52)	0.779
Tea (g/day)	−0.0004	−0.001 to −0.00001	0.046	Ref.	−14.60 (−51.96 to 22.76)	−44.22 (−84.21 to −4.24)	0.030

^a^ Models with DII as a continuous variable. DII was included into each model as an independent variable with diet parameters as dependent variables (one model for one dependent parameter); ^b^ Models with DII as a factorial parameter with 3 terciles (Tercile 1 as a baseline); ^c^ p-trend determined through the median approach; ^d^ All models adjusted for age, sex, place of living, marital status, education, smoking, alcohol, physical activity, and BMI. Coding of covariates was according to categories in Table 2.

**Table 5:** Comparison of anthropometric, biochemical, and atherogenic risk factors of 1791 participants of PURE Poland study population by dietary inflammatory index (DII) terciles.

Parameter		Total Group	Tercile 1	Tercile 2	Tercile 3	*p*	Post-Hoc
Systolic blood pressure, mmHg		145.32 ± 21.39	146.36 ± 21.21	145.30 ± 21.67	144.31 ± 21.27	0.253	
Diastolic blood pressure, mmHg		85.87 ± 11.24	86.06 ± 10.80	86.12 ± 11.65	85.43 ± 11.25	0.508	
Waist circumference, cm	Females	88.15 ± 13.53	87.35 ± 12.80	87.20 ± 13.29	89.90 ± 14.33	0.009	1, 2 < 3
	Males	99.23 ± 12.49	98.06 ± 11.97	99.53 ± 10.96	100.10 ± 14.27	0.206	
WHR	Females	0.84 ± 0.08	0.83 ± 0.07	0.83 ± 0.08	0.85 ± 0.08	<0.001	1, 2 < 3
	Males	0.96 ± 0.07	0.96 ± 0.08	0.96 ± 0.07	0.96 ± 0.08	0.858	
BMI, kg/m^2^		28.07 ± 5.06	28.23 ± 5.14	27.92 ± 4.81	28.06 ± 5.24	0.579	
TC, mmol/L		5.07 ± 1.00	5.05 ± 1.01	5.08 ± 0.99	5.08 ± 0.99	0.839	
TG, mmol/L		1.40 ± 0.74	1.32 ± 0.70	1.43 ± 0.78	1.43 ± 0.74	0.009	1 < 2, 3
HDL-C, mmol/L	Females	1.61 ± 0.40	1.62 ± 0.40	1.63 ± 0.40	1.59 ± 0.40	0.321	
	Males	1.33 ± 0.33	1.32 ± 0.28	1.30 ± 0.31	1.37 ± 0.37	0.089	
Fasting glucose, mg/dL		96.00 (88.00;105.00)	94.00 (87.00;104.00)	95.00 (88.00;105.00)	97.00 (89.00;107.00)	0.001	1, 2 < 3
LDL-C, mmol/L		2.92 ± 0.92	2.88 ± 0.91	2.93 ± 0.92	2.95 ± 0.92	0.423	
Atherogenic index of plasma (AIP)		0,28 ± 0.28	0,25± 0.28	0,30 ± 0.28	0,31 ± 0.28	<0.001	1 < 2, 3
Castelli’s Risk Index (CRI)		3.56 ± 1.09	3.42 ± 1.05	3.61 ± 1.08	3.66 ± 1.11	<0.001	1 < 2, 3

Data presented as mean ± SD or median (T1;T3), depending on data distribution. Tercile groups compared with ANOVA analysis or Kruskal–Wallis test. For ANOVA—post-hoc Tukey test was applied, for Kruskal–Wallis test—post-hoc Dunn test was applied. WHR—waist-hip ratio; BMI—body mass index; TC—total cholesterol; HDL-C—HDL cholesterol; LDL-C—LDL cholesterol; and TG—triglycerides.

## Data Availability

Data is contained within the article.
